# Colon perforation due to cytomegalovirus infection in a patient with idiopathic hypereosinophilic syndrome: a case report

**DOI:** 10.1186/s12876-020-01381-1

**Published:** 2020-07-23

**Authors:** Bin Luo, Chengxin Deng, Tieying Hou, Fangping Xu, Qianchao Liao, Yong Li, Junjiang Wang

**Affiliations:** 1 Department of Hematology, Guangdong Provincial People’s Hospital, Guangdong Academy of Medical Sciences, Guangzhou, 510080 China; 2 Division of Laboratory Medicine, Guangdong Provincial People’s Hospital, Guangdong Academy of Medical Sciences, Guangzhou, 510080 China; 3 Department of Pathology and Laboratory Medicine, Guangdong Provincial People’s Hospital, Guangdong Academy of Medical Sciences, Guangzhou, 510080 China

**Keywords:** Colon perforation, Cytomegalovirus infection, Idiopathic hypereosinophilic syndrome, Immunosuppression therapy, Case report

## Abstract

**Background:**

Hypereosinophilic syndrome (HES) is a very rare disease and usually treated with corticosteroids. Gastrointestinal (GI) cytomegalovirus (CMV) infection is also rare but frequent in patients with immunocompromised status. These two related diseases present with similar manifestations, and may result in a life-threatening complication: perforation. However, the treatment strategies differ greatly. Here, we report a case of colon perforation due to cytomegalovirus infection in a patient with idiopathic HES.

**Case presentation:**

A 41-year-old man with a history of HES was transferred to our hospital due to an acute onset of abdominal pain. During the treatment course of HES, this patient received CMV-DNA test with a result of < 2000 copies/ml. Computed tomography (CT) suggested colon perforation. An emergency surgery was performed immediately. Pathological diagnosis revealed CMV infection and infiltration of eosinophils. This patient received both anti-CMV therapy and immunosuppression therapy. Subsequently, the patient recovered and was discharged 25 days after the operation.

**Conclusion:**

During the course of HES treatment, CMV infection should be reconsidered if digestive symptoms relapse.

## Background

Hypereosinophilia (HE) is defined as an absolute count of peripheral blood eosinophils greater than 1500/mm^3^, and it may be associated with tissue injury [1]. Hypereosinophilic syndrome (HES) is a very rare disease with a reported age-adjusted incidence rate of 0.036 per 100,000 [2]. The first-line treatment for HES is steroids. Cytomegalovirus (CMV) is a double-stranded DNA virus in the herpesvirus family [3, 4]. The CMV infection rate in the general population varies from 40 to 100% [5–7]. However, initial infection of CMV is frequently asymptomatic, and it may become latent for life in immunocompetent hosts. Symptomatic gastrointestinal cytomegalovirus (GI-CMV) infection is rare, but common among patients in immunocompromised status [8]. These two related diseases can present with similar symptoms of abdominal pain, diarrhea, and GI bleeding. However, the treatment strategy for these two diseases differs greatly. Incorrect medications may result in life-threatening complications. Here, we report a case of colon perforation due to CMV infection in a patient with HES.

## Case presentation

**Fig. 1:**
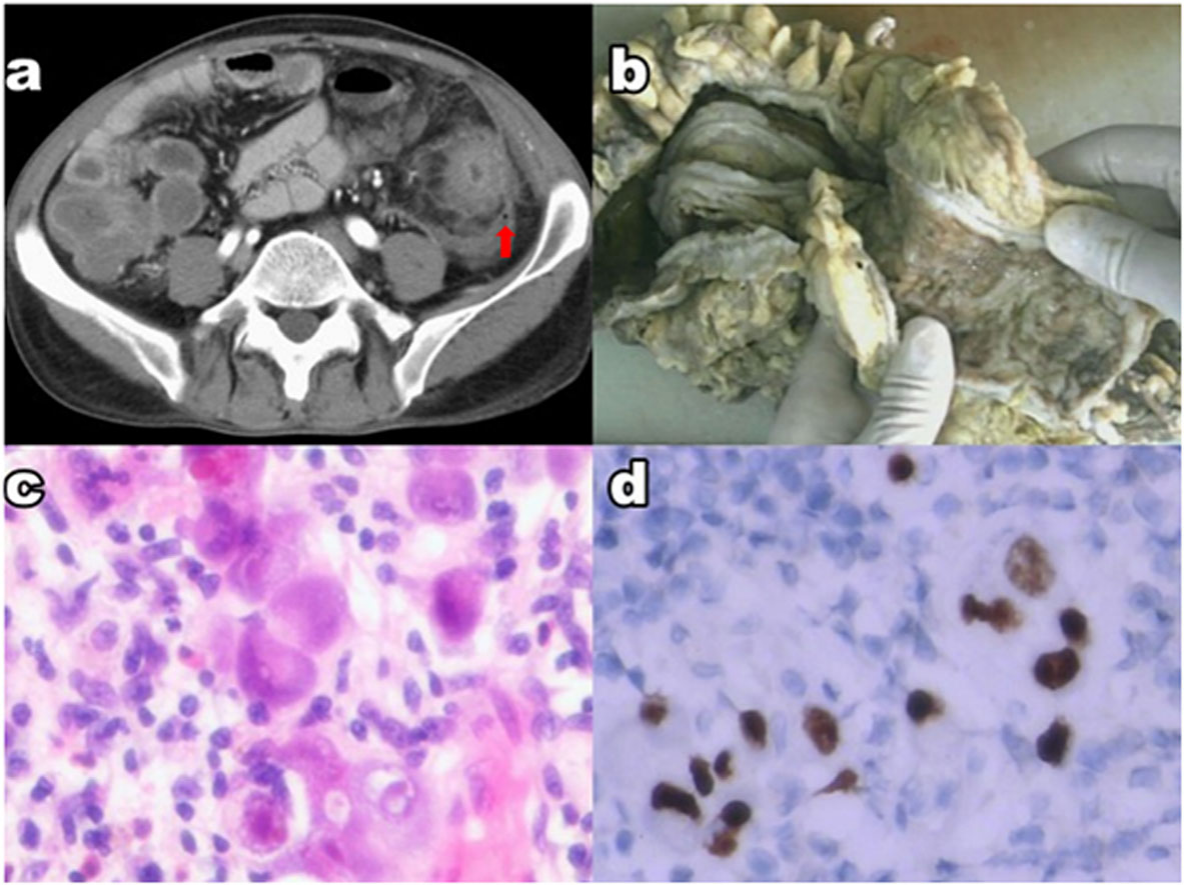
**a** Swollen descending colon wall and free air (red arrow). **b** Swollen bowel wall, deep ulcer and mucosal erosion on specimen. **c** Massive virus inclusion body. **d** Immunohistochemical staining confirmed the infection of CMV

The patient was a 41-year-old Asian man, who suffered from diarrhea and skin itch 4 years ago and was diagnosed with HES. He received pharmacotherapy of methylprednisolone. The initial dosage was 40 mg per day and was gradually reduced to 28 mg per day. During the course of immunosuppression therapy, the patient received CMV-DNA test with a result of < 2000 copies/ml. This patient was transferred to our hospital due to an acute onset of abdominal pain for 3 h. He denied a history of parasite infection. Stool-rt was performed and ova weren’t found. There was no history of drug or food allergies or any other significant medical history. The physical examination found skin rashes over the entire body and typical signs of peritonitis. Laboratory examinations showed increased CRP levels of 102 mg/L. The complete blood cell count revealed a white blood cell count of 27.93*10^9^/L with a differential of 60.0% eosinophils. The total level of IgE was greater than 2000 IU/ml. Abdominal contrast enhanced CT revealed that the descending colon wall was swollen and free air existed (Fig. 1a). The patient underwent an emergency operation of left hemicolectomy immediately. Swollen bowel wall, multiple deep ulcers and mucosal erosions were found in specimens (Fig. 1b). Pathological diagnosis revealed massive virus inclusion bodies (Fig. 1c) and infiltration of eosinophils, but without any evidence of thrombus, vasculitis or occlusion. Immunohistochemical staining confirmed CMV infection (Fig. 1d). CMV-DNA test was performed again postoperatively and the copy number of CMV in blood was 8.02*10^4^/ml. The patient received Ganciclovir (5 mg/kg, twice daily for 21 days) therapy, and dexamethasone (5 mg per day) was also taken to control the symptoms of HES. Finally, the patient was discharged from our hospital 25 days after the operation. The CMV-PCR test results were lower than 2000 copies per milliliter, and the percentage of eosinophils decreased to the normal range.

## Discussions and conclusions

**Fig. 2:**
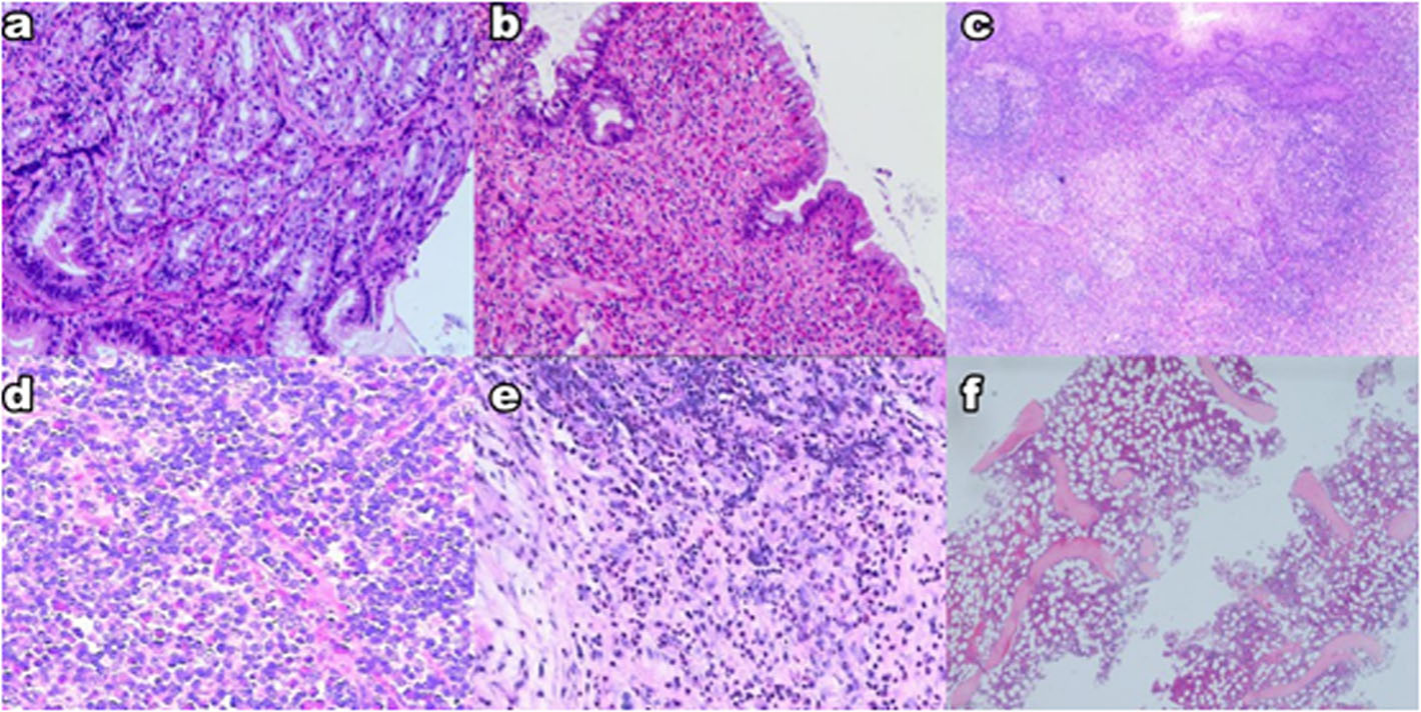
Massive eosinophil infiltration into multiple organs. **a** Stomach. **b** Colon. **c** Tonsil. **d** Cervical lymph node. **e** Periorchium. **f** Bone marrow

To make a diagnosis of HES, the absolute count of eosinophils > 1500/mm^3^ must persist longer than 6 months, tissue damage must be present, and other diseases must be excluded [1, 9] (Table 1). Theoretically, all organs are susceptible to the infiltration of sustained eosinophilia. Dermatologic involvement is the most common (69%), followed by pulmonary (44%) and gastrointestinal (38%) involvement [10]. In our case, multiple organs were infiltrated by eosinophils, including the stomach, colon, tonsil, cervical lymph nodes and perididymis (Fig. 2).

**Table 1:** Diagnosis of idiopathic hypereosinophilic syndrome (HES)

Exclusion of the following diseases	
1. Reactive eosinophilia	
2. Lymphocyte-variant hypereosinophilia (cytokine-producing, immunophenotypically-aberrant T-cell population)	
3. Chronic eosinophilic leukemia, NOS	
4. WHO-defined myeloid malignancies associated eosinophilia (e.g. MDS, MPNs, MDS/MPNs, or AML)	
5. Eosinophilia-associated MPNs or AML/ALL with rearrangements of PDGFRA, PDGFRB, or FGR1.	

*Abbreviations*: *NOS* not other specified, *MDS* myelodysplastic syndrome, *MPNs* myeloproliferative neoplasms, *AML* acute myeloid leukemia, *ALL* acute lymphoblastic leukemia, *PDGFRA* platelet-derived growth factor receptor alpha, *PDGFRB* platelet-derived growth factor receptor beta, *FGR1* fibroblast growth factor receptor 1

The essential goal of the therapy is to alleviate eosinophil-mediated organ damage. Corticosteroids are the first-line therapy for patients with symptoms and organ involvement. Hydroxyurea and interferon-alpha have demonstrated efficacy as initial treatment and steroid-refractory cases [1]. Long-term corticosteroid treatment may suppress patients’ immune systems, making them susceptible to microorganisms.

Latent CMV infection may become active in the presence of acute stressful conditions or in the case of severe or prolonged immunosuppression, for example, HIV-positive patients and transplant recipients. The GI tract is the most frequent site infected by CMV [11, 12]. It is characteristic to find multiple ulcers during endoscopy in GI-CMV which can result in gastrointestinal perforation, which is life-threatening once it occurs.

HES, the utilization of corticosteroids and GI-CMV infection can damage the gastrointestinal mucosa and cause perforation. Two cases of digestive tract perforation associated with hypereosinophilic syndrome (HES) have been reported. And digestive tract perforation was attributed to acute thrombosis [13, 14]. In this case, because pathological examination revealed a massive CMV inclusion body and a few infiltration of eosinophils, but without any evidence of thrombus, vasculitis or occlusion, we concluded that acute colon perforation resulted from cytomegalovirus infection after immunosuppression therapy.

Abdominal pain, diarrhea and GI bleeding are the manifestations of both HES and CMV infection, but the treatment strategies for these two diseases differ greatly from each other. In this case, when the symptoms of abdominal pain, diarrhea and GI bleeding relapsed, the dosage of corticosteroids was increased. However, these symptoms were actually caused by CMV infection. Incorrect immunosuppression therapy will exacerbate the infection of CMV, and lead to colon perforation.

The ‘gold standard’ assay for CMV is the CMV-antigenemia (AG) test with a reported sensitivity and specificity of 70–90% [15]. Because the levels of CMV-AG are proportional to disease severity and treatment response, the CMV-AG test can be utilized as an indicator for monitoring active CMV infection and guiding treatment [16]. CMV-PCR is a new method to detect active CMV infection which has comparable sensitivity and specificity [17, 18]. In this case, the onset of abdominal pain, diarrhea during immunosuppression therapy may suggest that latent CMV infection became active. However, the optimal treatment time was missed. Hence, if digestive symptoms relapse during the course of HES treatment, CMV infection should be reconsidered even though the latest result of CMV-DNA test was negative.

## Data Availability

The datasets used and/or analysed during the current study are available from the corresponding author on reasonable request.

## Abbreviations

HES: Hypereosinophilic syndrome
GI: Gastrointestinal
CMV: Cytomegalovirus
CT: Computed tomography
AG: Antigenemia
CRP: C-reaction protein
PDGFRA: Platelet-derived growth factor receptor alpha
PDGFRB: Platelet-derived growth factor receptor beta
FGR1: Fibroblast growth factor receptor 1
